# Supplementary material for: Novel Driver Strength Index highlights important cancer genes in TCGA PanCanAtlas patients
Source: PeerJ. 2022 Aug 11;10:e13860. doi: 10.7717/peerj.13860 (PMC9375969; doi:10.7717/peerj.13860)
Supplement: Supplemental Information 2 [file peerj-10-13860-s002.zip › COHORTS/genes plots/detailed/2021_8_16_14_9_distribution_events_detailed_9.pdf]

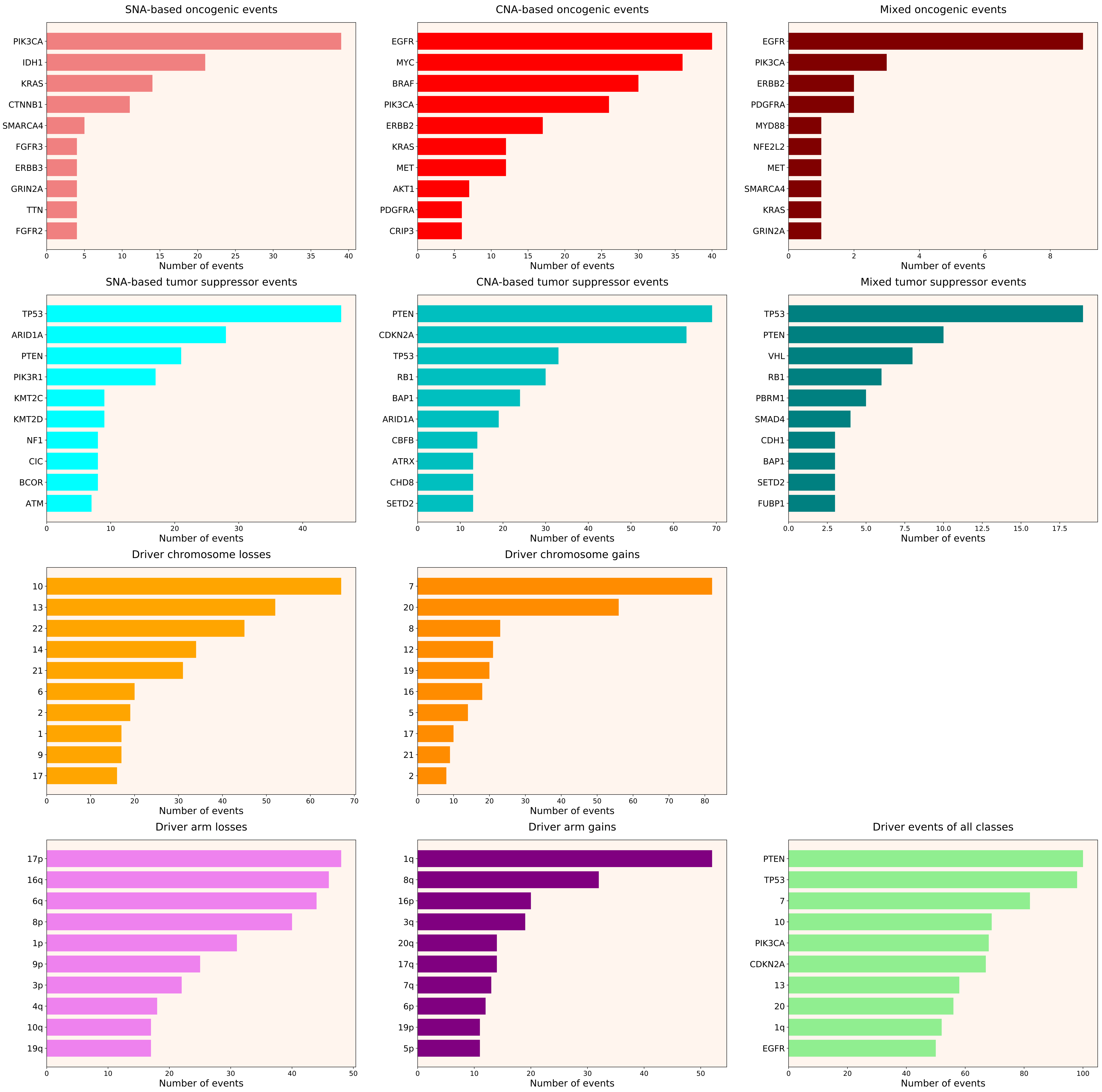
SNA-based oncogenic events
PIK3CA
IDH1
KRAS
CTNNB1
SMARCA4
FGFR3
ERBB3
GRIN2A
TTN
FGFR2
Number of events
CNA-based oncogenic events
EGFR
MYC
BRAF
PIK3CA
ERBB2
KRAS
MET
AKT1
PDGFRA
CRIP3
Number of events
Mixed oncogenic events
EGFR
PIK3CA
ERBB2
PDGFRA
MYD88
NFE2L2
MET
SMARCA4
KRAS
GRIN2A
Number of events
SNA-based tumor suppressor events
TP53
ARID1A
PTEN
PIK3R1
KMT2C
KMT2D
NF1
CIC
BCOR
ATM
Number of events
CNA-based tumor suppressor events
PTEN
CDKN2A
TP53
RB1
BAP1
ARID1A
CBFB
ATRX
CHD8
SETD2
Number of events
Mixed tumor suppressor events
TP53
PTEN
VHL
RB1
PBRM1
SMAD4
CDH1
BAP1
SETD2
FUBP1
Number of events
Driver chromosome losses
10
13
22
14
21
6
2
1
9
17
Number of events
Driver chromosome gains
7
20
8
12
19
16
5
17
21
2
Number of events
Driver arm losses
17p
16q
6q
8p
1p
9p
3p
4q
10q
19q
Number of events
Driver arm gains
1q
8q
16p
3q
20q
17q
7q
6p
19p
5p
Number of events
Driver events of all classes
PTEN
TP53
7
10
PIK3CA
CDKN2A
13
20
1q
EGFR
Number of events
